# Supplementary material for: Tumour microbiomes and Fusobacterium genomics in Vietnamese colorectal cancer patients
Source: NPJ Biofilms Microbiomes. 2022 Oct 29;8:87. doi: 10.1038/s41522-022-00351-7 (PMC9616903; doi:10.1038/s41522-022-00351-7)
Supplement: Supplementary file 1 — Supplementary Information [file 41522_2022_351_MOESM1_ESM.pdf]

Tumour microbiomes and *Fusobacterium* genomics in Vietnamese colorectal cancer patients

Supplementary Information

Supplementary Figures

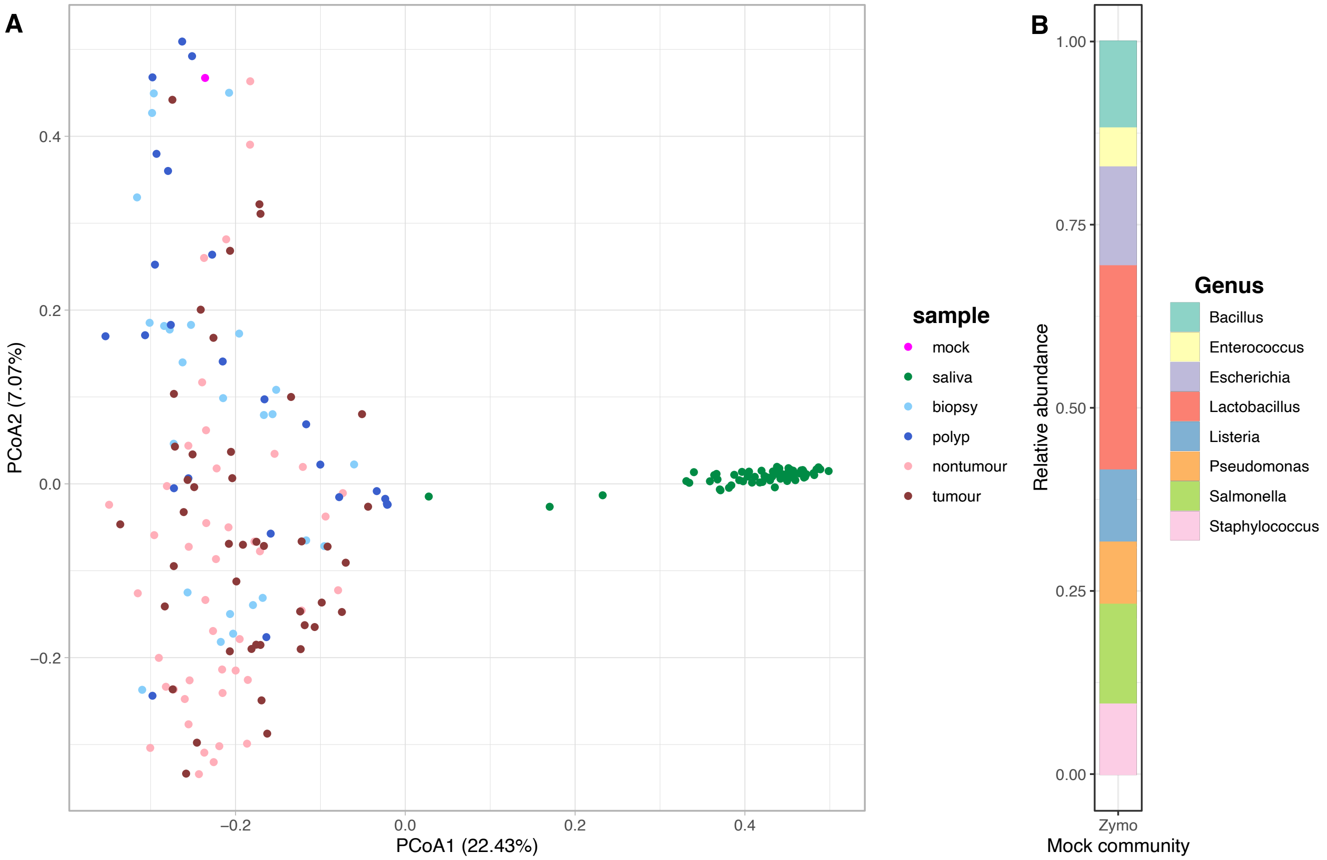

Supplementary Figure 1 Quality check of 16S rRNA sequencing experiment (A) Principal coordinate analysis (PCoA) conducted on Bray-Curtis distance, with samples coloured by sample types. (B) Taxonomic composition of the positive control – Zymo mock community.

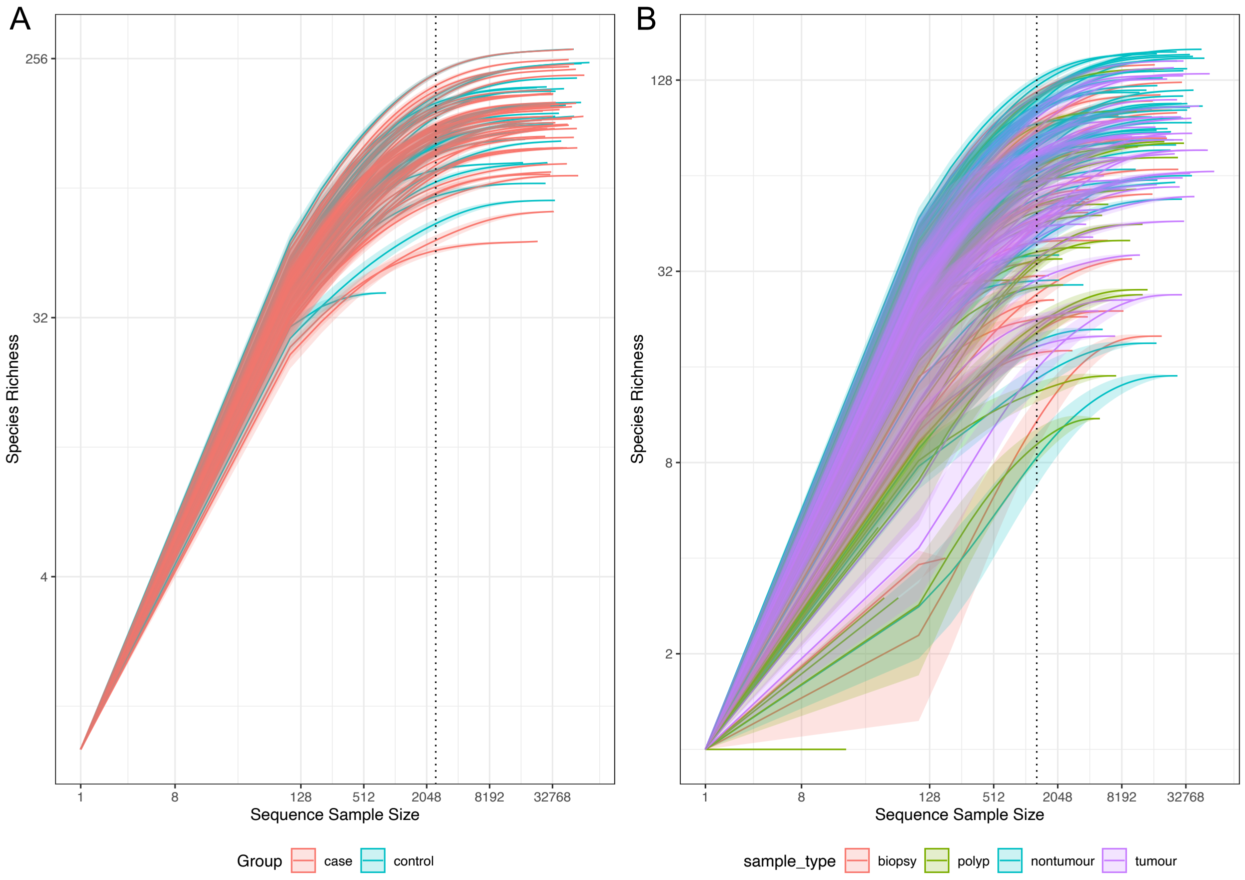

Supplementary Figure 2 Rarefaction curves outlined for (A) the salivary microbiomes and (B) the gut mucosal microbiomes. Rarefaction curves were constructed using the "ggrare" function (step size = 100), with both axes plotted in log2 of the calculated values. The vertical dotted lines represent the thresholds (2,500 for (A) and 1,300 for (B)), below which samples with low sequencing depth were removed. The shaded area represents the standard errors associated with the mean calculated species richness value in each line.

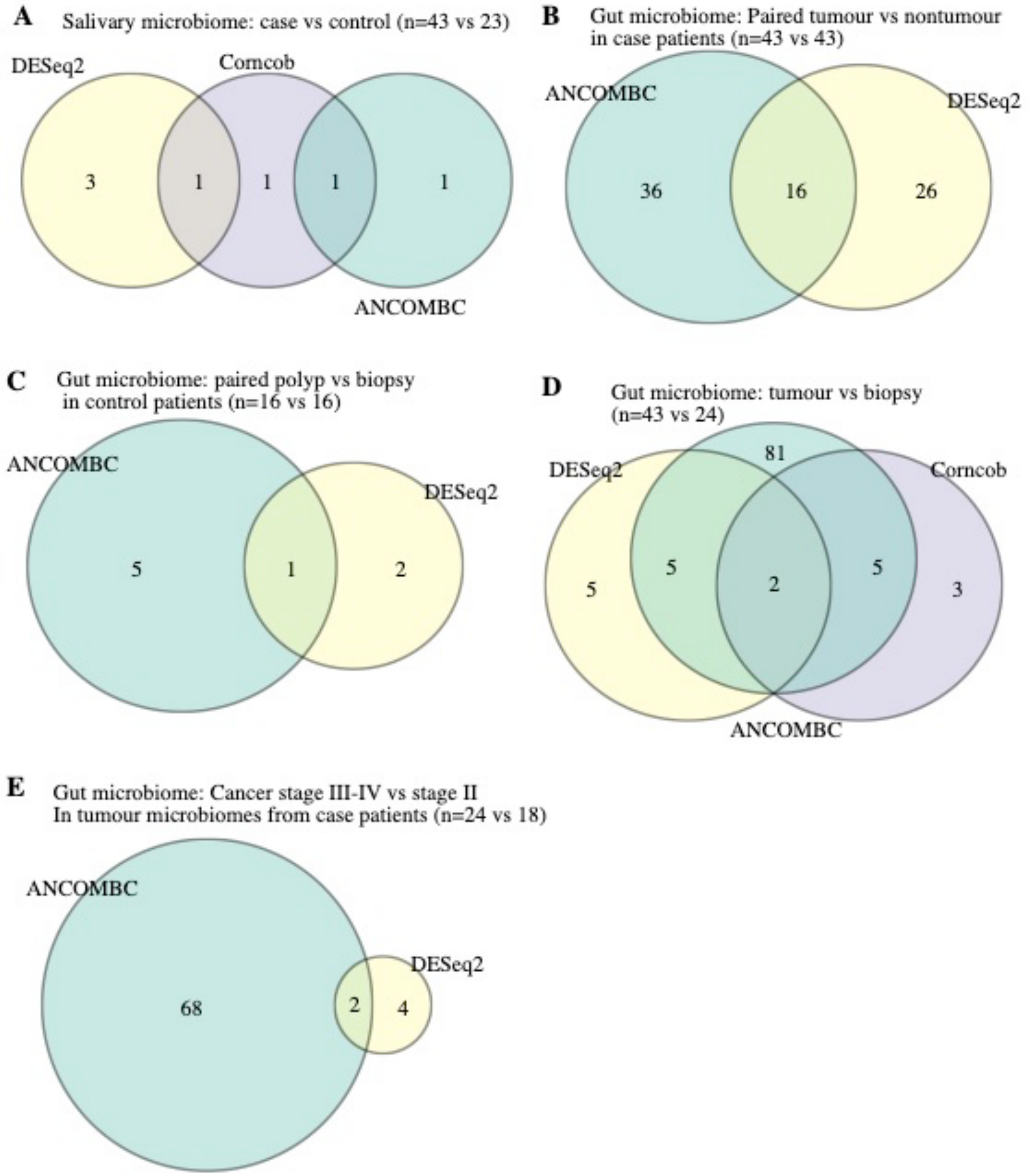

Supplementary Figure 3 Summary of differential abundance analysis results in different comparison (A) salivary microbiome between cases and controls; Gut microbiomes between (B) paired tumour and non-tumour in CRC cases, (C) paired polyp and biopsy in controls, (D) tumour (cases) and biopsy (control), (E) cancer stage III-IV and stage II in tumours from CRC cases.

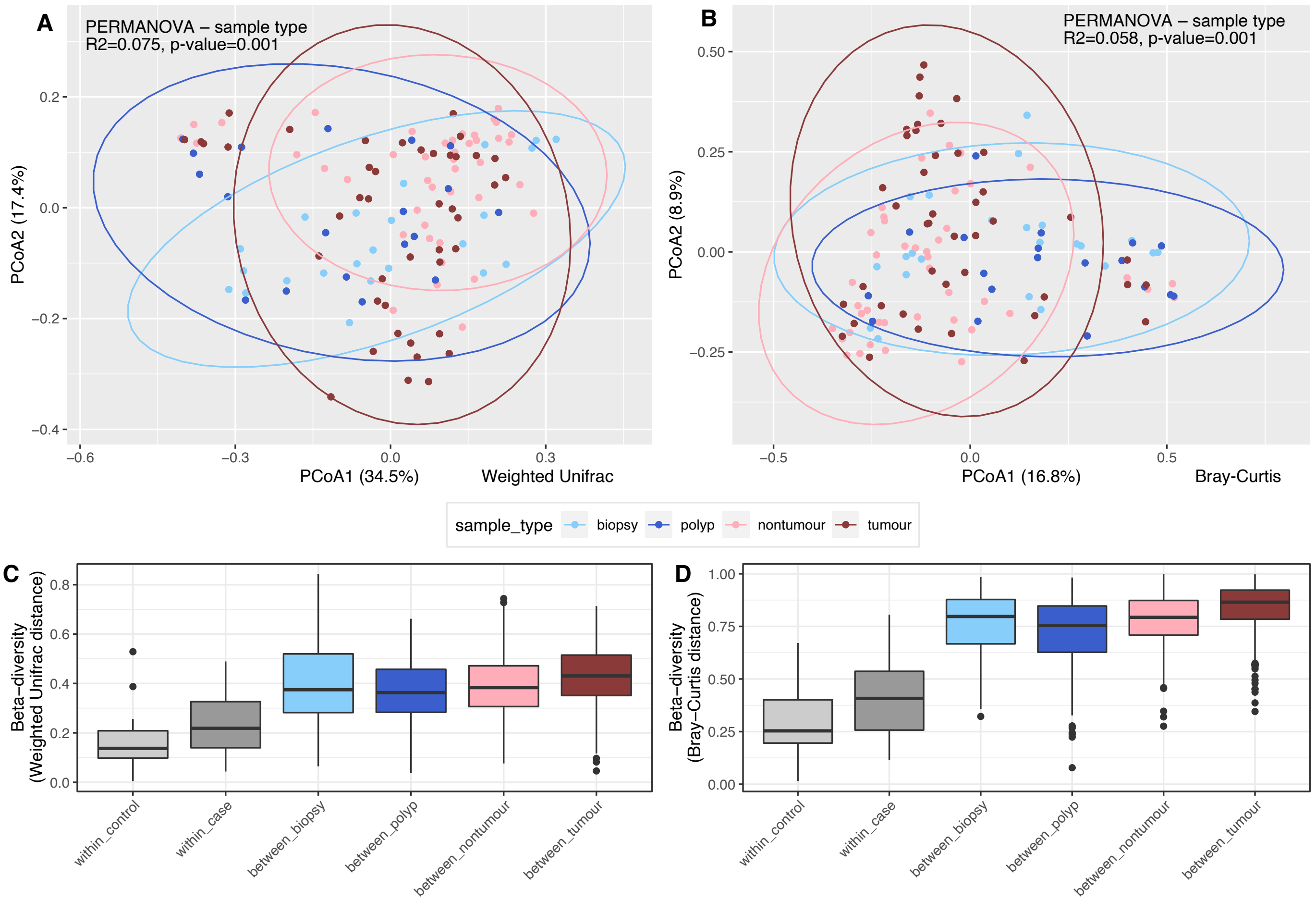

Supplementary Figure 4 The gut mucosal microbiomes of colorectal cancer patients in this study. Principal coordinate analyses (PCoA) of 129 gut mucosal microbiomes, conducted on (A) Weighted Unifrac distance, and (B) Bray-Curtis distance, with colours denoting different sample types. Boxplots showing the distribution of pairwise beta-diversity observed in each gut microbiome category, calculated using the (C) Weighted Unifrac distance, and (D) Bray-Curtis distance.

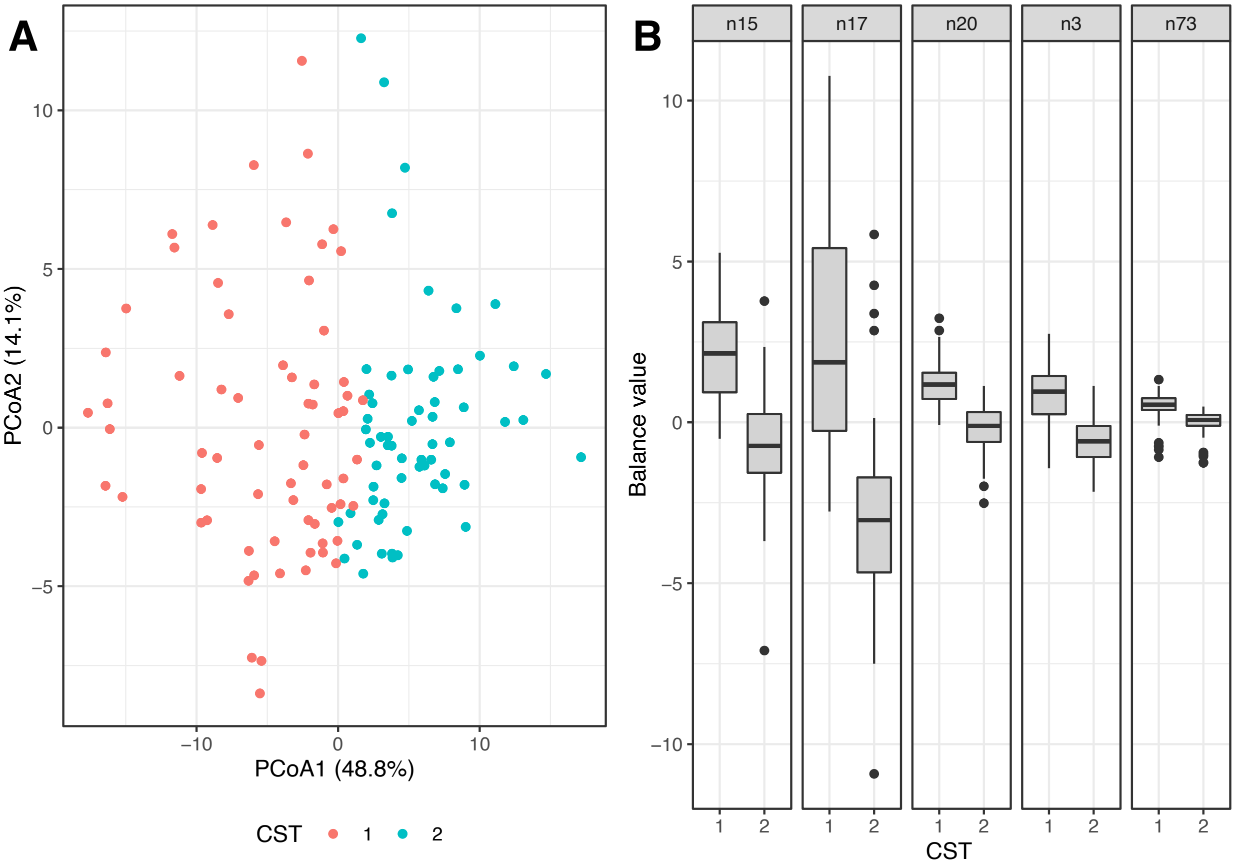

Supplementary Figure 5 Clustering of the gut mucosal microbiomes into two community state types (CSTs). (A) Principal coordinate analysis (PCoA) conducted on phylogenetic-assisted isometric log-ratio (PhILR) transformed data (similar to Figure 1B), with samples coloured by CST membership. (B) “Balances” identified by Random forest classification as most important in differentiating the two CSTs, with values shown in boxplot for each CST. N15: Actinobacteria+Proteobacteria/Firmicutes+Fusobacteria, n17: Proteobacteria/Actinobacteria, n20: Gammaproteobacteria/Campilobacterota+Desulfobacterota; n3: other bacteria/Lachnospiraceae, n73: *Escherichia*/*Morganella*. Source data are provided as a Source Data file.

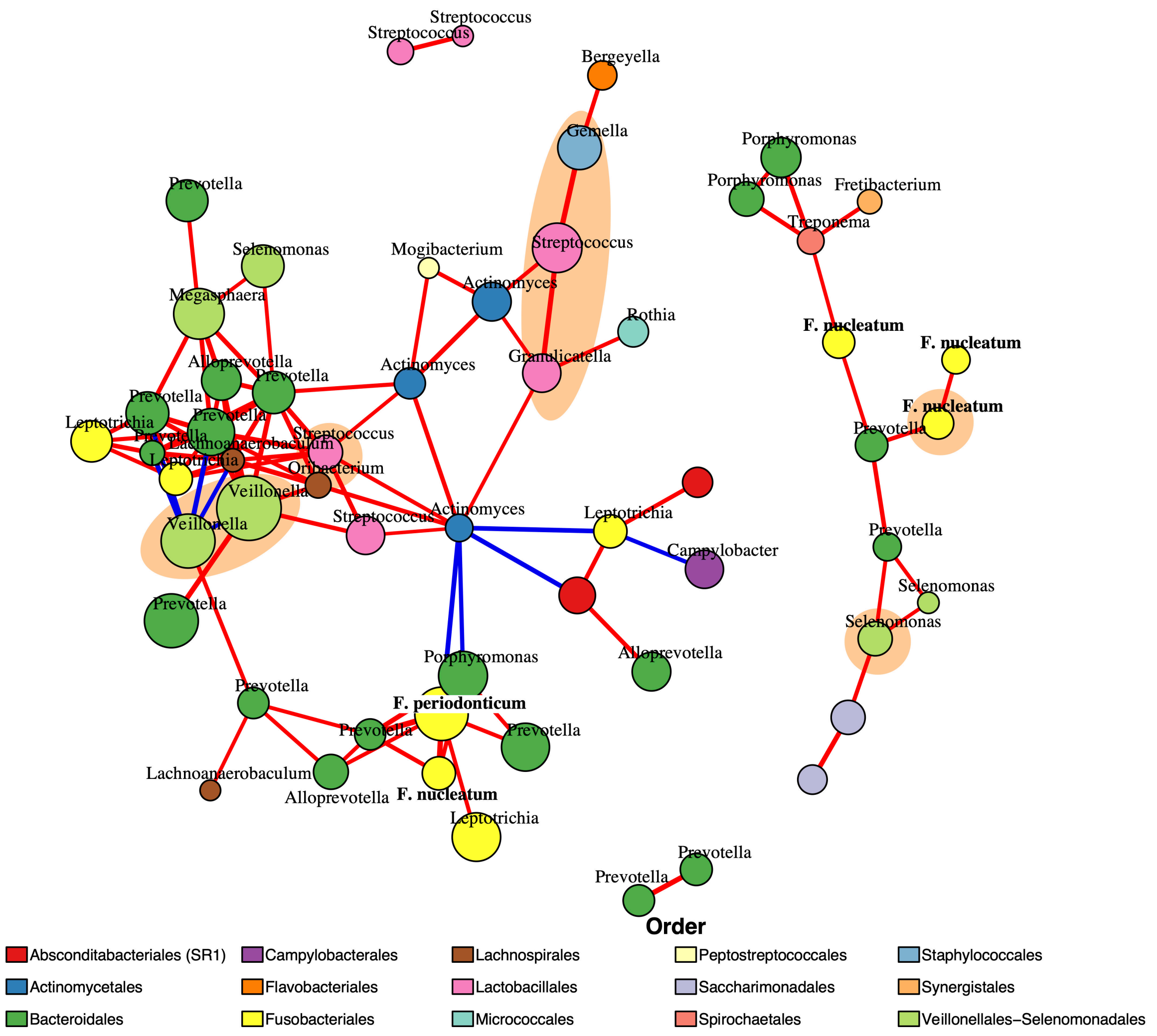

Supplementary Figure 6 Correlation network of salivary microbiomes from all participants. The network was constructed from 115 most representative ASVs sampled from 66 mucosal microbiomes, outlining significant interactions detected by both CCLasso (p value ≤ 0.01 and absolute correlation strength > 0.4) and SpiecEasi. Positive and negative interactions were coloured as red and blue lines respectively, with line weight proportional to correlation strength. The ASVs (nodes) were coloured based on taxonomic family (see Legend), with sizes proportional to their relative abundances. The orange shaded area entails ASVs detected in the gut mucosal microbiome correlation network of CRC patients (see Figure 3). Source data are provided as a Source Data file.

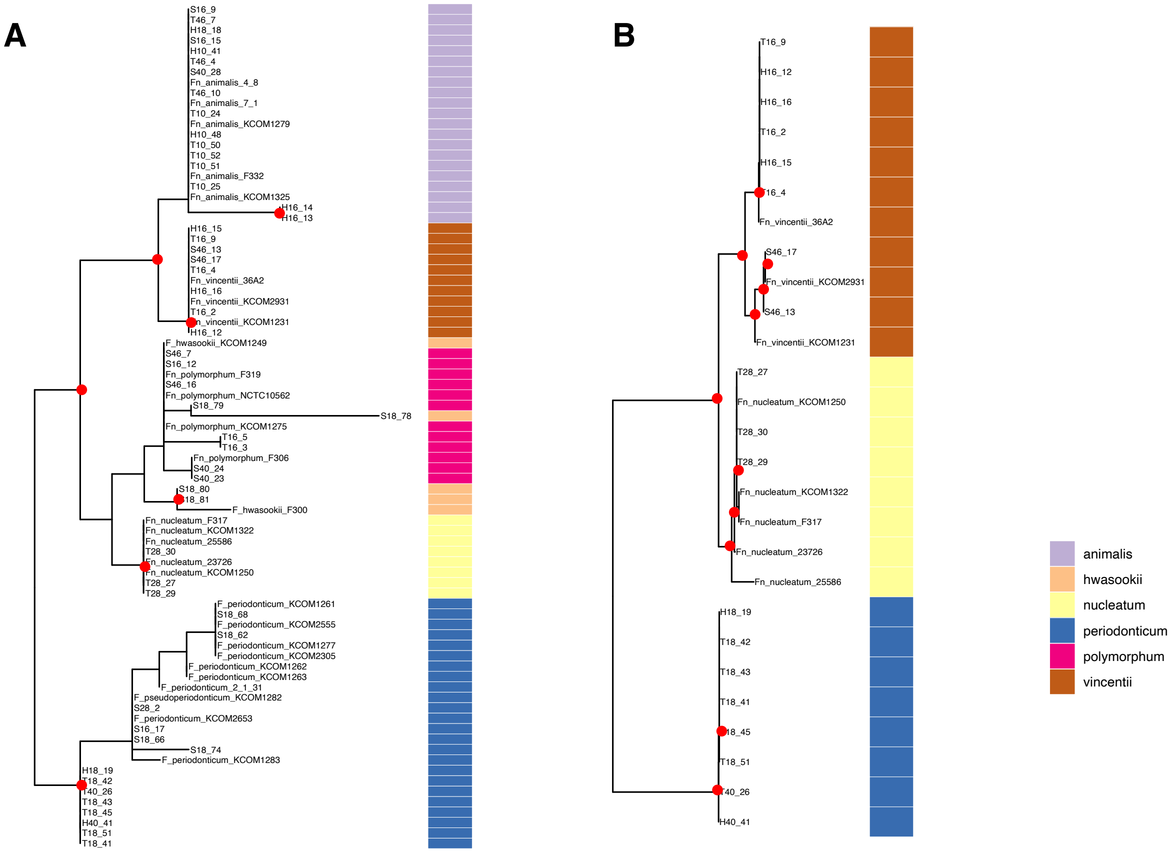

Supplementary Figure 7 Maximum likelihood phylogenies of (A) FadA and (B) CbpF proteins. Red circles at internal nodes denote bootstrap values ≥ 70. The column on the right denotes different *Fusobacterium* species/subspecies.

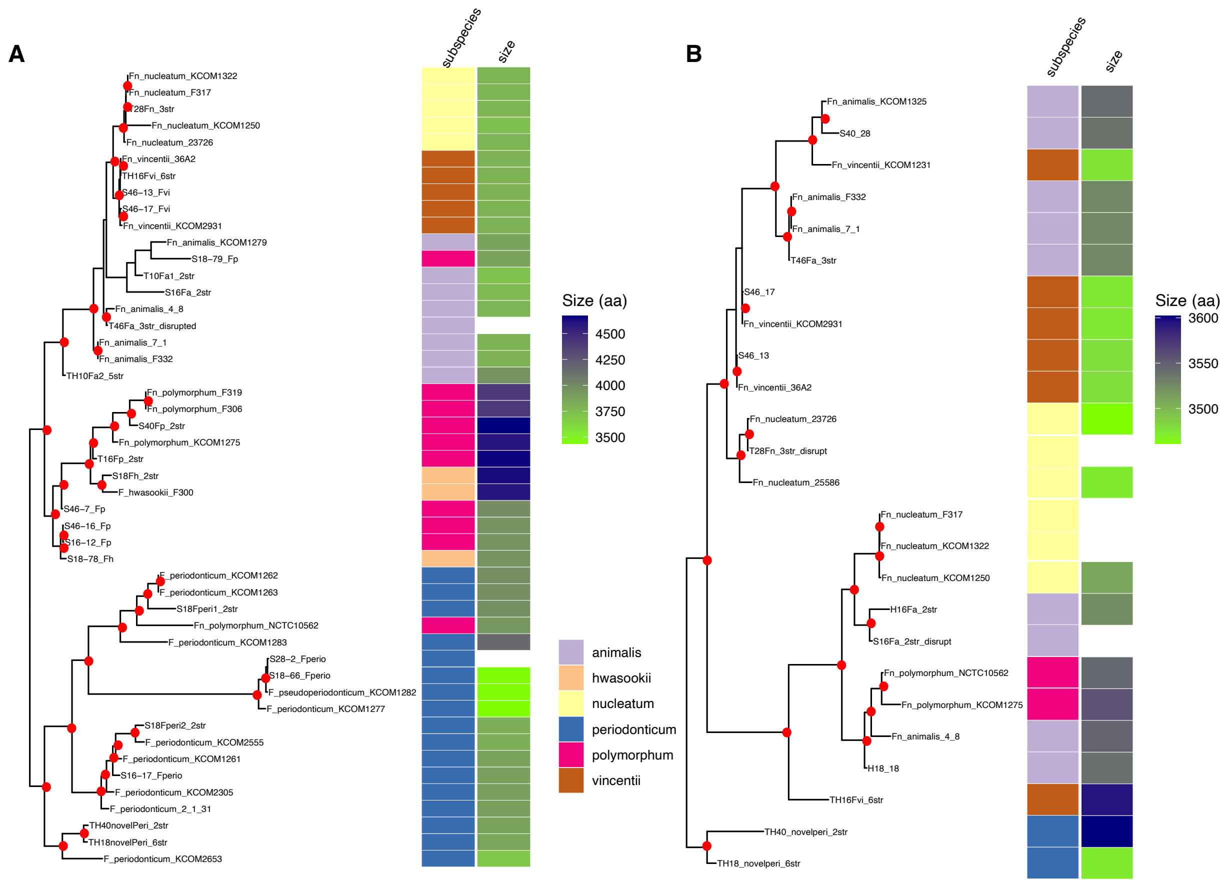

Supplementary Figure 8 Maximum likelihood phylogenies of (A) Fap2 and (B) RadD proteins. Red circles at internal nodes denote bootstrap values ≥ 70. The columns on the right denote different *Fusobacterium* species/subspecies and the size of each protein. Disrupted proteins were left blank in size.
